# Genomic and phenotypic insight into *Xanthomonas vesicatoria* strains with different aggressiveness on tomato

**DOI:** 10.3389/fmicb.2023.1185368

**Published:** 2023-06-27

**Authors:** María Isabel Bianco, María Agustina Ponso, Jerson Garita-Cambronero, Valeria Paola Conforte, Tadeo E. Galván, Germán Dunger, Gustavo M. Morales, Adrián Alberto Vojnov, Ana María Romero, Jaime Cubero, Pablo Marcelo Yaryura

**Affiliations:** ^1^Instituto de Ciencia y Tecnología Dr. César Milstein – Fundación Pablo Cassará – Consejo Nacional de Investigaciones Científicas y Técnicas (CONICET), Buenos Aires, Argentina; ^2^Instituto de Investigación en Medicina y Ciencias de la Salud, Facultad de Medicina, Universidad del Salvador, Buenos Aires, Argentina; ^3^Instituto Multidisciplinario de Investigación y Transferencia Agroalimentario y Biotecnológica (IMITAB, UNVM-CONICET), Instituto Académico Pedagógico de Ciencias Básicas y Aplicadas, Universidad Nacional de Villa María, Villa María, Argentina; ^4^Asociación Nacional de Obtentores Vegetales (ANOVE), Madrid, Spain; ^5^Facultad de Ciencias Agrarias, Instituto de Ciencias Agropecuarias del Litoral, CONICET, Universidad Nacional del Litoral, Esperanza, Argentina; ^6^Departamento de Química, Facultad de Ciencias Exactas, Físico-Químicas y Naturales, Instituto de Investigaciones en Tecnologías Energéticas y Materiales Avanzados, Universidad Nacional de Rio Cuarto – CONICET, Rio Cuarto, Argentina; ^7^Cátedra de Fitopatología, Departamento de Producción Vegetal, Facultad de Agronomía, Universidad de Buenos Aires, Buenos Aires, Argentina; ^8^Laboratorio de Bacteriología, Departamento de Protección Vegetal, Instituto Nacional de Investigación y Tecnología Agraria/Consejo Superior de Investigaciones Científicas (INIA/CSIC), Madrid, Spain

**Keywords:** *Xanthomonas vesicatoria*, bacterial spot, xanthan, bacterial motility, type IV pili, flagellum, tomato

## Abstract

*Xanthomonas vesicatoria* is one of the causal agents of bacterial spot, a disease that seriously affects the production of tomato (*Solanum lycopersicum*) and pepper (*Capsicum annum*) worldwide. In Argentina, bacterial spot is found in all tomato producing areas, with *X. vesicatoria* being one of the main species detected in the fields. Previously, we isolated three *X. vesicatoria* strains BNM 208, BNM 214, and BNM 216 from tomato plants with bacterial spot, and found they differed in their ability to form biofilm and in their degree of aggressiveness. Here, the likely causes of those differences were explored through genotypic and phenotypic studies. The genomes of the three strains were sequenced and assembled, and then compared with each other and also with 12 other publicly available *X. vesicatoria* genomes. Phenotypic characteristics (mainly linked to biofilm formation and virulence) were studied *in vitro*. Our results show that the differences observed earlier between BNM 208, BNM 214, and BNM 216 may be related to the structural characteristics of the xanthan gum produced by each strain, their repertoire of type III effectors (T3Es), the presence of certain genes associated with c-di-GMP metabolism and type IV pili (T4P). These findings on the pathogenicity mechanisms of *X. vesicatoria* could be useful for developing bacterial spot control strategies aimed at interfering with the infection processes.

## 1. Introduction

Bacterial spot is one of the most destructive diseases that affect tomato (*Solanum lycopersicum* L.) and pepper (*Capsicum annuum* L.) worldwide. The four lineages of *Xanthomonas* that cause bacterial spot are *Xanthomonas euvesicatoria*, *Xanthomonas vesicatoria*, *Xanthomonas gardneri and Xanthomonas perforans* (Jones et al., 2004; Potnis et al., 2015; Constantin et al., 2016). Strains belonging to different physiological races have been described within each of these four species, according to their pathogenic relationship with tomato and/or pepper plants (Sahin and Miller, 1998; Gore and O'Garro, 1999). More specifically, the races of xanthomonads that caused bacterial spot are recognised based on the hypersensitive response (HR) that they trigger on tomato and pepper lines (Jones et al., 2004). Different strains can infect tomato, pepper, or both. Strains that can infect tomato are classified into races T1-T5 and those that can infect pepper are classified into races P0-P10 (Astua-Monge et al., 2000; Jones et al., 2004; Stall et al., 2009). T1 strains correspond to *X. euvesicatoria* and carry the *avrRxv* effector (Whalen et al., 1993). T2 strains belong to *X. vesicatoria* given that they do not contain the avirulence genes *rx1*, *rx2* and *rx3*, *Xv3* or *Xv4*; they do not induce HR in any of the tomato indicator cultivars. Races T3, T4 and T5 tend to be *X. perforans*. T3 strains carry the *avrXv3* effector which induces HR in the Xv3-containing genotype and T4 strains have *avrXv4* which induces HR in the *Xv4*-containing genotype. Only *S. pennellii* x Hawaii 7998 provides resistance to race T5 (Thind, 2019). In pepper, differential genotypes carrying the *Bs1*, *Bs2*, *Bs3* or *Bs4* genes interact with the avirulence genes *avrBs1*, *avrBs2*, *avrBs3*, and *avrBs4*, respectively, that are present in bacterial strains (Cook and Guevara, 1984; Gaxiola, 2000). Recently, *X. euvesicatoria* and *X. perforans* were reclassified as two pathovars of the same species (*X. euvesicatoria* pv. *euvesicatoria* and *X. euvesicatoria* pv. *perforans*) and *X. gardneri* was reclassified as *X. hortorum* pv. *gardneri,* while the taxonomic classification of *X. vesicatoria* did not change (Osdaghi et al., 2021). *X. vesicatoria* frequently causes bacterial spot in field grown tomatoes in Argentina (Romero et al., 2003).

Previously, we found significant differences in the severity of bacterial spot caused by three *X. vesicatoria* strains (BNM 208, BNM 214, and BNM 216) isolated from diseased tomato plants in Argentina. These strains also showed differences in their motility, adhesion to surface, ability to form biofilm and in the viscosity of the xanthan they produce (Felipe et al., 2018). Although these factors are likely to play an essential role in the ability of *X. vesicatoria* to successfully colonise and infect tomato, identification of other critical virulence factors is needed for a deeper understanding of the pathogenesis.

The virulence factors that have been described for *Xanthomonas* so far include adhesins, flagella, fimbriae/pili, exopolysaccharides (EPSs), lipopolysaccharides (LPSs), different secretion systems and their effectors, extracellular degrading enzymes, biofilm formation, and a regulatory network involved in the coordination of these elements (An et al., 2020). Many of these virulence factors and bacterial structures have been widely studied in some phytopathogenic *Xanthomonas* spp., but little is known about some of them in *X. vesicatoria*. As in other xanthomonads, the type III secretion system (T3SS) in *X. vesicatoria* is particularly important for virulence, since it manipulates plant processes for the benefit of the pathogen by introducing type III effectors (T3Es) into the cytosol of the host cell (Thieme et al., 2005; Lorenz et al., 2008; Büttner, 2016). On the other hand, an important aspect of many pathogenic bacteria, including also *Xanthomonas* spp., is their ability to form biofilms to survive and interact with their hosts (Castiblanco and Sundin, 2016). The development of biofilms allows bacteria to resist biotic and abiotic stresses, supports the coordination of adaptive responses to environmental changes, and enables bacterial multiplication under stressful conditions (Picchi et al., 2021). A prerequisite for biofilm formation is the attachment to a surface through structures of a broad group of fimbrial and non-fimbrial adhesins (Felipe et al., 2018).

Flagella and T4P are multiprotein structures involved in bacterial motility, attachment, adhesion, biofilm formation and pathogenesis (Kearns, 2010; Burdman et al., 2011; Duan et al., 2013; Haiko and Westerlund-Wikström, 2013; Wood, 2013), whose importance has been demonstrated in some phytopathogenic *Xanthomonas* spp. (Rigano et al., 2007a; Malamud et al., 2011; Bianco et al., 2016; Dunger et al., 2016). T4P, in particular, are implicated in natural transformation, phage infection, and twitching motility (Caserta et al., 2010; Burrows, 2012; Dunger et al., 2014, 2016). There are different types of T4P, which are divided into two major subclasses, type IVa and IVb, though only type IVa pilins mediate twitching motility (Burrows, 2012). For its assembly and function, T4P require numerous structural and regulatory components, as major pilins, which are named PilA (main component of the filament), and minor pilins, which are present in lower quantities and prime the assembly of T4P (Nguyen et al., 2015; Ronish et al., 2019). PilA proteins belong to the type IVa pilins subclass, characterized by having a small prepilin leader sequence (5–6 amino acids) in the N-terminal region containing the G-1F + 1/E+5 motif. This motif is required for the cleavage of the prepilin leader sequence (between G-1 and F + 1) and the subsequent methylation of the phenylalanine located at the N-terminal end of the mature protein. Both modifications are essential to incorporate the mature pilin subunits into the nascent pilus (Dunger et al., 2014).

On the other hand, xanthan gum is the main EPS synthesised by *Xanthomonas* spp. and an essential component of the extracellular biofilm matrix. It contributes to epiphytic survival before plant colonisation and plays an important role in biofilm formation, being necessary for bacterial virulence and disease progression (Dow et al., 2003; Yun et al., 2006; Torres et al., 2007; Rigano et al., 2007a,b; Aslam et al., 2008; Darsonval et al., 2009; Yaryura et al., 2014; Bianco et al., 2016). Earlier, we reported the effects of xanthan composition, structure and viscosity on the characteristics of biofilm and virulence in *Xanthomonas campestris* pv. *campestris* (Xcc) (Bianco et al., 2016) and *Xanthomonas citri* subsp. *citri* strain 306 (Xac306) (Conforte et al., 2019). Moreover, we observed that xanthan-deficient mutants were unable to form biofilm (Rigano et al., 2007a; Bianco et al., 2016).

*Xanthomonas* genomes from all named *Xanthomonas* spp. are currently available in public databases and have been used in studies addressed to different purposes, including those aimed to elucidate virulence or host range factors in this bacterial genus (Timilsina et al., 2020). Since 2011 genome sequencing projects from bacterial spot of tomato and pepper xanthomonads have been generated and applied in studies addressed to explain the relationship among species and pathovars, their virulence and host range basis, the diversity of effectors and their epidemiology (Potnis et al., 2011; Schwartz et al., 2015; Barak et al., 2016). Our work is aimed to analyse bacterial virulence factors that might explain the differences in the aggressiveness between three *X. vesicatoria* strains isolated from Argentina (Felipe et al., 2018), on the basis of comparative phenotypic and genotypic analysis. Our goal is to increase our knowledge on virulence factors of *X. vesicatoria* that could be useful in other *Xanthomonads* models, as a preliminary step towards future new disease control methods.

## 2. Materials and methods

### 2.1. Bacterial strains and culture conditions

The three *X. vesicatoria* strains used in this work were BNM 208, BNM 214, and BNM 216. They had been isolated from leaves of tomato plants with symptoms of bacterial spot from commercial field crops and deposited at the culture collection of the “Banco Nacional de Microorganismos” (BNM, Argentina, WDCM number 938, GCM) as described previously (Felipe et al., 2018).

The strains were routinely grown on modified yeast dextrose calcium carbonate (YDC) agar plates (Ritchie and Dittapongpitch, 1991) or in peptone-yeast extract-malt extract (PYM) medium (Cadmus et al., 1976) and incubated at 28°C for 24–72 h, depending on the experiment and with shaking at 200 r.p.m. for liquid cultures.

### 2.2. Genomic sequencing and annotation

The DNA of the strains was extracted from 30 mL of a pure and fresh bacterial culture using the Wizard® Genomic DNA Purification Kit Promega (Madison, WI, United States) according to the manufacturer’s instructions. DNA quantity and quality were determined by Picodrop microlitre Spectrophotometer (Picodrop Ltd., Cambridge, United Kingdom) and through 0.8% agarose gel electrophoresis. The genomes of BNM 208, BNM 214, and BNM 216 strains were sequenced using the Illumina HiSeq 2,500 platform (2 × 100 bp paired-end reads) by an external sequencing service (Macrogen Inc., NGS Service, Seoul, Korea). The quality of the obtained reads was examined by FastQC v.0.11.8 (Andrews et al., 2010). Raw reads were quality filtered and trimmed with Trimmomatic v.0.38 (Bolger et al., 2014) (Parameters: ILLUMINACLIP:TruSeq3-PE.fa:2:30:10, LEADING:3 TRAILING:3 SLIDINGWINDOW:4:15 MINLEN:36). *De novo* genome assemblies of BNM 208, BNM 214, and BNM 216 were performed with MIRA v.4.0 (Chevreux et al., 1999) and some quality parameters (Table 1), such as the number of contigs, the finish quality (according to BUSCO completeness), N50, GC %, were verified by QUAST v.4.5 using its web version with default parameters (Gurevich et al., 2013). Automatic annotation of the draft genome sequences was conducted using the Prokka v.1.13.3 pipeline (Seemann, 2014) using a specific database constructed with all the complete reference genome sequences of the genus *Xanthomonas* available at NCBI, as well as using the Prokaryotic Genome Annotation Pipeline (PGAP) available at NCBI (Tatusova et al., 2016). The presence of signal peptides (leader sequences), transmembrane domains and the assignment of a potential function according to the Gene Ontology (GO) and the Clusters of Orhtologous Groups of proteins (COG) databases (Tatusov et al., 2001; The Gene Ontology Consortium, 2021) were performed for all the protein-coding sequences by using signalP v.5.0 (Almagro Armenteros et al., 2019), the TMHMM server v.2.0 (Krogh et al., 2001) and the functional annotation pipelines available in the OmicsBox (v. 1.3), the NCBI CD-Search online platform (Lu et al., 2020) and the Geneious (v. 7.1) software by using default parameters.

**Table 1:** Sequencing information and genome statistics of *X. vesicatoria* strains BNM 208, BNM 214 and BNM 216.

Property/attribute	BNM 208	BNM 214	BNM 216
Total number of reads	21,312,890	24,589,638	25,173,242
Q20/Q30 (%)	95.42/88.81	95.42/88.81	95.42/87.97
Finishing quality	Draft	Draft	Draft
BUSCO completeness score	99.5%	99.9%	99.7%
Library construction	TruSeq DNA PCR-free sample preparation kit	TruSeq DNA PCR-free sample preparation kit	TruSeq DNA PCR-free sample preparation kit
Sequencing platform	Illumina HiSeq	Illumina HiSeq	Illumina HiSeq
Number of contigs	115	26	20
Fold coverage	98x	98x	98x
N_50_ (bp)	178,916	740,125	541,440
Assembler	MIRA 4.0	MIRA 4.0	MIRA 4.0
Genome annotation	Prokka v.1.13.3 NCBI-PGAP	Prokka v.1.13.3 NCBI-PGAP	Prokka v.1.13.3 NCBI-PGAP
Locus tag	G8D12	G8D19	G8D20
GenBank Accession No	PRJNA610021	PRJNA610030	PRJNA610032
Genome size (bp)	5,142,907	5,424,305	5,302,342
DNA G + C (%)	64.5	64	64.1
Total genes	4,454	4,671	4,569
Protein coding sequences	4,335	4,570	4,467
RNA genes	4 rRNA, 62 tRNA	3 rRNA, 53 tRNA	3 rRNA, 54 tRNA
Genes with predicted functions	3,686	3,768	3,707
Genes assigned to COGs	3,157	3.162	3,126
Genes with signal peptide	876	932	908
Genes with transmembrane helices	1,321	1.367	1,363
Plasmids	pLMG911.2	pLM159.1 plCMP7383.3	pLMG911.1 pLMG911.2

Genomic sequences of the three *X. vesicatoria* strains studied in this article were deposited in the GenBank database: GenBank Accession N° PRJNA610021 (BNM 208), PRJNA610030 (BNM 214) and PRJNA610032 (BNM 216).

### 2.3. Comparative genome analysis

Output data obtained from Prokka was used as input data to confirm the taxonomy of the sequenced strains by calculating the Average Nucleotide Identity (ANI) based on the BLAST algorithm compared to the reference genome sequences of the *Xanthomonas* species. These data were also used to perform the pangenomic analysis by comparing the genome of the three sequenced strains with other 12 *X. vesicatoria* genome sequences publicly available (Supplementary Table S1). These two analyses were conducted with the pipelines in Pyani v.0.2.7 (Pritchard et al., 2016) and Roary v.3.11.2 (Page et al., 2015).

A batch of 410 genes associated with pathogenesis in *Xanthomonas* (TBDTs, TonB dependent transporters; STCRs, sensors of the two-component regulatory system; MCPs, methyl accepting chemotaxis proteins; structure and regulation of flagella and T4P, production of xanthan gum, quorum sensing, cell wall degrading enzymes, secretion systems – types II, III, and IV – as well as their associated effectors) (Garita-Cambronero et al., 2019), was searched in all the 15 available genome sequences of *X. vesicatoria* with BLASTp by using an identity and coverage cutoff of 80%. Finally, the presence of plasmids was ascetained with the PLSDB plasmid database (Galata et al., 2019).

### 2.4. Bioinformatics analysis of genes associated with T4P

The identity of each sequence within the T4P cluster was confirmed with the protein databases InterPro (Blum et al., 2020) and BLASTp, and the online ORF Finder server (Open Reading Frame Finder; RRID:SCR_016643). In addition, the online server iPro54-PseKNC (Lin et al., 2014) was used to identify the presence of σ^54^ promoters as binding sites for the transcriptional regulator PilR. To identify the presence of the signal sequence characteristic of type IVa pilins, the protein sequences found were aligned using the ClustalW tool in MEGA11 software (Molecular Evolutionary Genetics Analysis version 11; Tamura et al., 2021).

### 2.5. Polymerase chain reaction (PCR) assays

The bacterial genomic DNA was prepared with Wizard® Genomic DNA Purification Kit (Promega Corporation, Madison, United States). PCR amplifications were carried out in a T-18 thermocycler (Ivema Desarrollos, Argentina). The samples contained PCR buffer 1X, 200 μM of each deoxynucleoside triphosphate (Inbio Highway, Tandil, Argentina), 100 mM of one primer set (see Supplementary Table S2), 1.5 mM of MgCl_2_, 1.25 U of Taq DNA polymerase (PB-L, Productos Bio-Lógicos SA, Buenos Aires, Argentina) and 50 ng of pure genomic DNA preparation in a total volume of 20 μL. The samples were heated as follows: 94°C for 3 min, 30 cycles at 94°C for 30 s, annealing temperature (of each primer set) for 30 s, 72°C for 1 min, and a final cycle at 72°C for 5 min. The PCR products were analysed by electrophoresis on 1.5% (w/vol) agarose gels.

### 2.6. Gene expression analysis

The strains were cultured in PYM medium until the stationary phase. The cells were harvested at OD_600_ 1–1.2. Total RNA was extracted with TRIzol (Gibco-BRL, Burlington, ON, Canada) following the manufacturer’s instructions, and quantified by spectrometry. Its integrity was checked by agarose gel electrophoresis. Reverse transcription was conducted by using random primers and M-MLV RT Promega (Madison, WI, United States). All primers used in this work were designed with the software Primer Express 3.0 (Applied Biosystems, Foster City, CA, USA) (Supplementary Table S2). The reactions were performed using SybrGreen master mix (Roche, Mannheim, Germany) and a Step One Real Time-PCR system (qPCR) (Applied Biosystems), as described previously (Conforte et al., 2019). The protocol for the qPCR was as follows: 50°C for 2 min, initial denaturation at 95°C for 5 min, followed by 40 cycles of 10 s at 95°C and 30 s at 60°C. The qPCR data analysis and primer efficiencies were obtained using LinReg PCR software (Ramakers et al., 2003). The 16S rRNA gene was used to standardise the expression of a given target gene; then, a ratio between treatments was calculated using the algorithm developed by Pfaffl et al. (2002). The relative expression ratios and the statistical analyses were performed using the *fgStatistics* software interface (Di Rienzo, 2012). The cutoff for statistically significant differences was set at *p* < 0.05.

### 2.7. Motility assays

Swimming and swarming motility assays were performed as described by Picchi et al. (2016). In both cases, 2 μL of bacterial suspensions of BNM 208, BNM 214, and BNM 216 strains (OD_600_ 0.1) were placed in the centre of Petri dishes with PYM medium with 0.5% (w/v) agar and NYGB medium with 0.25% (w/v) agar for swarming and swimming assays, respectively. After incubation at 28°C for 72 h, swimming and swarming motility of each strain was quantified by measuring the diameter of the bacterial growth halo with Image J1.4 software (Wayne Rasband, National Institutes of Health, United States).^1^

### 2.8. Examination of flagella

Flagellum staining for subsequent observation by optical microscopy was performed as previously described in Kodaka et al. (1982). Briefly, the staining solution was prepared by mixing 10 parts of the mordant solution [2 g tannic acid, 10 mL 5% (w/v) phenol, 10 mL saturated aqueous of AlKO_8_S_2_.12H_2_O] with one part of the crystal violet solution [12% (w/v) in ethanol]. A drop of an overnight culture of each strain was placed on a glass slide and air dried. Then, samples were stained with 10 mL of staining solution and observed at 1,000× with Leica DM1000 LED (Leica, Wetzlar, Germany) optical microscope. Images were taken with Leica MC170 HD camera.

### 2.9. Twitching motility: time-lapse imaging

T4P dependent movement (twitching motility) was studied by monitoring the position of bacterial cells under the microscope at different time intervals, following Llontop et al. (2021) with some modifications. Briefly, the different strains were grown on LB agar plates at 28°C, and then some colonies were sampled by scraping the plates. Bacterial suspensions were prepared with sterile water, and 2 μL of each were placed on a King B agar slab, left to dry, and covered with a coverslip. After 30 min at 28°C, they suspensions were observed under a Leica DM1000 LED (Leica, Wetzlar, Germany) microscope (1,000×), and images were taken every 30 s with a Leica MC170 HD camera.

The King-B agar plates were prepared as described by Bayer-Santos et al. (2019) with slight modifications. Briefly, the top of a glass slide was completely covered with double-sided tape (clear 3 M VHB, 19 mm wide, 1 mm thick). A rectangular space in the center was cut with a scalpel, removed and filled with melted King B medium containing 1% (w/v) agar and supplemented with 2 mM of CaCl_2_. To create a flat and smooth surface, a second glass slide was placed over it and was carefully slipped off when the medium solidified.

### 2.10. Xanthan analysis by atomic force microscopy (AFM)

AFM, which is based on the interaction between an exploratory tip and the surface of a sample (in our case, xanthan), makes it possible to visualize individual well separated macromolecules on a mica surface (Ikeda et al., 2012). Xanthan gum produced by the three *X. vesicatoria* strains was obtained as described previously described by Felipe et al. (2018). For the AFM analysis, xanthans were dispensed into type I grade water (Purelab Classic, Elga Veolia) at a concentration of *ca.* 5–15 μg mL^−1^. A drop of each sample was deposited onto a freshly cleaved mica (V-1 Grade, SPI Suppliers), kept in contact with the surface for 1–2 min, then the excess was removed by a gentle tilt of the substrate and left to dry in air.

The AFM experiments were performed with an Agilent Technologies SPM/AFM microscope model 5500, which was mounted into a vibration isolation chamber (Agilent Technologies Inc.) and operated in air at room temperature. The samples were analysed under intermittent contact (Acoustic AC Mode) using silicon cantilevers (MikroMasch, NSC15/Al BS) with a spring constant in the range of 20–75 N/m, oscillating at frequencies just below the resonance. The tip was scanned on the sample surface by A-type open-loop Multi-purpose Scanner (90 × 90 μm^2^) at a speed of 0.4–2 lines/s depending on the size of the observed area. The topographical images were created from raw data values treated and analysed using Gwyddion 2.60^2^ software package, corrected only by using the 2D levelling (usually plane level) and scan line corrections (usually align rows) tools.

### 2.11. *In planta* assays

Bacterial suspensions of *X. vesicatoria* strains BNM 208, BNM 214, and BNM 216 [10^8^ colony-forming unit (CFU) mL^−1^] were infiltrated into the abaxial leaflets and leaf surfaces of differential tomato differential plants using a needless syringe. The tomato differential lines used were *S. lycopersicum* Bonny Best (universal susceptible line), Hawaii 7998 (effective against *avrRxv*; race T1) (Whalen et al., 1993) and *Solanum pimpinellifolium* plant introduction (PI) 128216 (effective against *avrXv3*; race T3) (Scott et al., 1995).

### 2.12. Statistical analysis

Three replicate trials were carried out for each sample, and all the experiments were repeated three times. Data from experiments were analysed by one-way ANOVA and mean differences were determined by Tukey’s test at a significance level of *p ≤ 0.05*. For ANOVA, parametric assumptions were tested using the Shapiro–Wilk test for normality, and the Levene’s test for homogeneity of variance using the R functions of the R package stats. All statistical analyses were performed using the INFOSTAT 1.0 software (Di Rienzo et al., 2009). Data from the experiments were expressed as mean ± the standard deviation (SD).

## 3. Results

### 3.1. Genome sequencing and comparative analysis of *Xanthomonas vesicatoria* strains BNM 208, BNM 214, and BNM 216

#### 3.1.1. General features

The whole-genomes of *X.vesicatoria* strains BNM 208, BNM 214, and BNM 216 were sequenced to explore genomic characteristics that might explain their different phenotypes. Table 1 shows information and statistics of the sequencing project, as well as the main general genomic features. The genome assemblies, based on 21,212,892 to 25,173,242 usable reads, resulted in 115 contigs for BNM 208, 26 for BNM 214, and only 20 for BNM 216, all with 98-fold coverage. Despite the difference in contig numbers among the strains, a similar number of genes and protein-coding sequences (CDS) was predicted (Table 1). Additionally, the potential plasmid sequences found in all three genomes were more than 99% identical to what had been previously described in *X. vesicatoria* and *X. gardneri* (Table 1).

**Figure 1:**
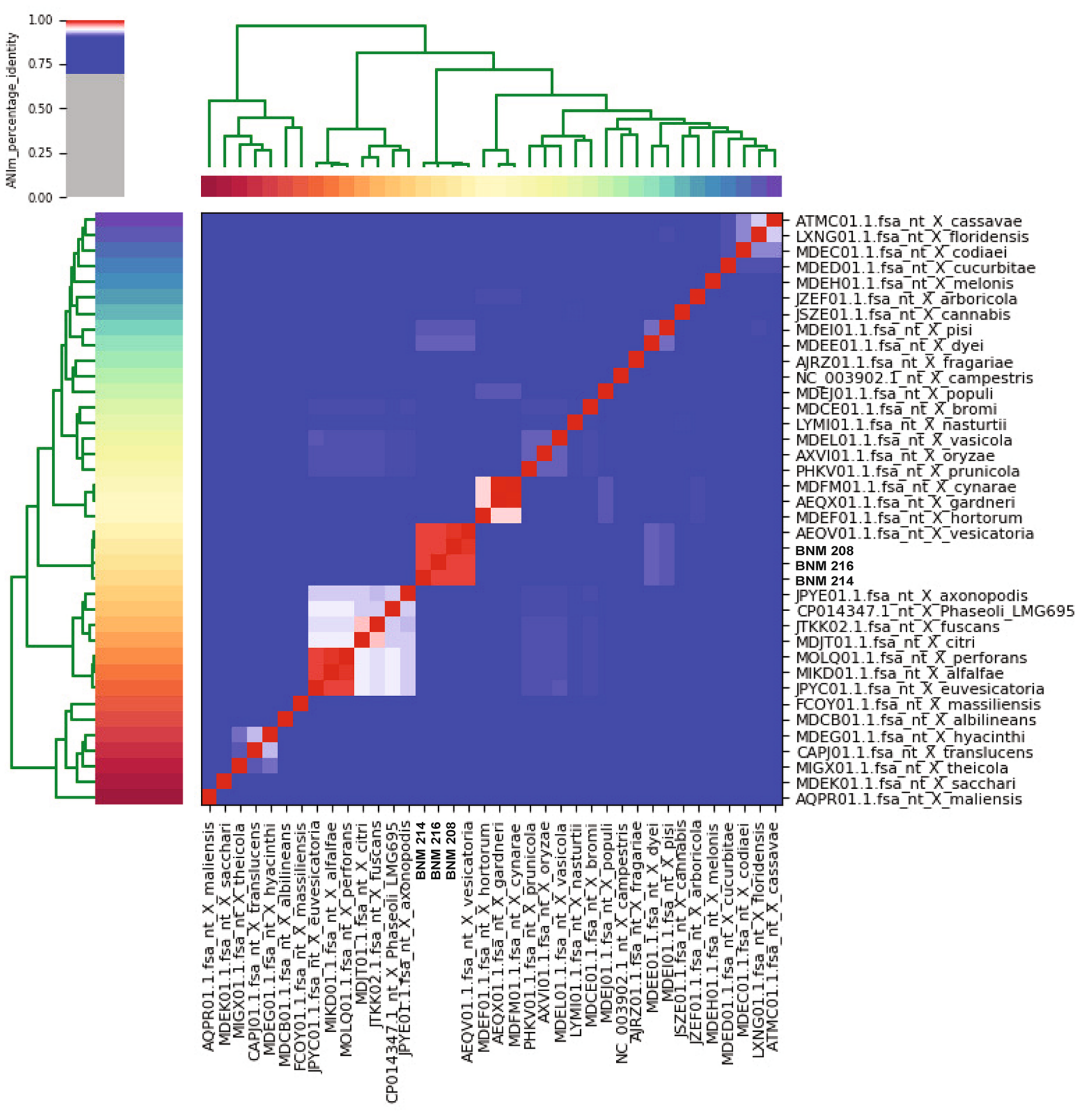
Average nucleotide identity (ANI) analysis. Confirmation of the taxonomy of BNM 208, BNM 214, and BNM 216 by calculating ANI compared to the reference genome sequences of the *Xanthomonas* species. The analysis shows ANI percentage over 99%.

**Figure 2:**
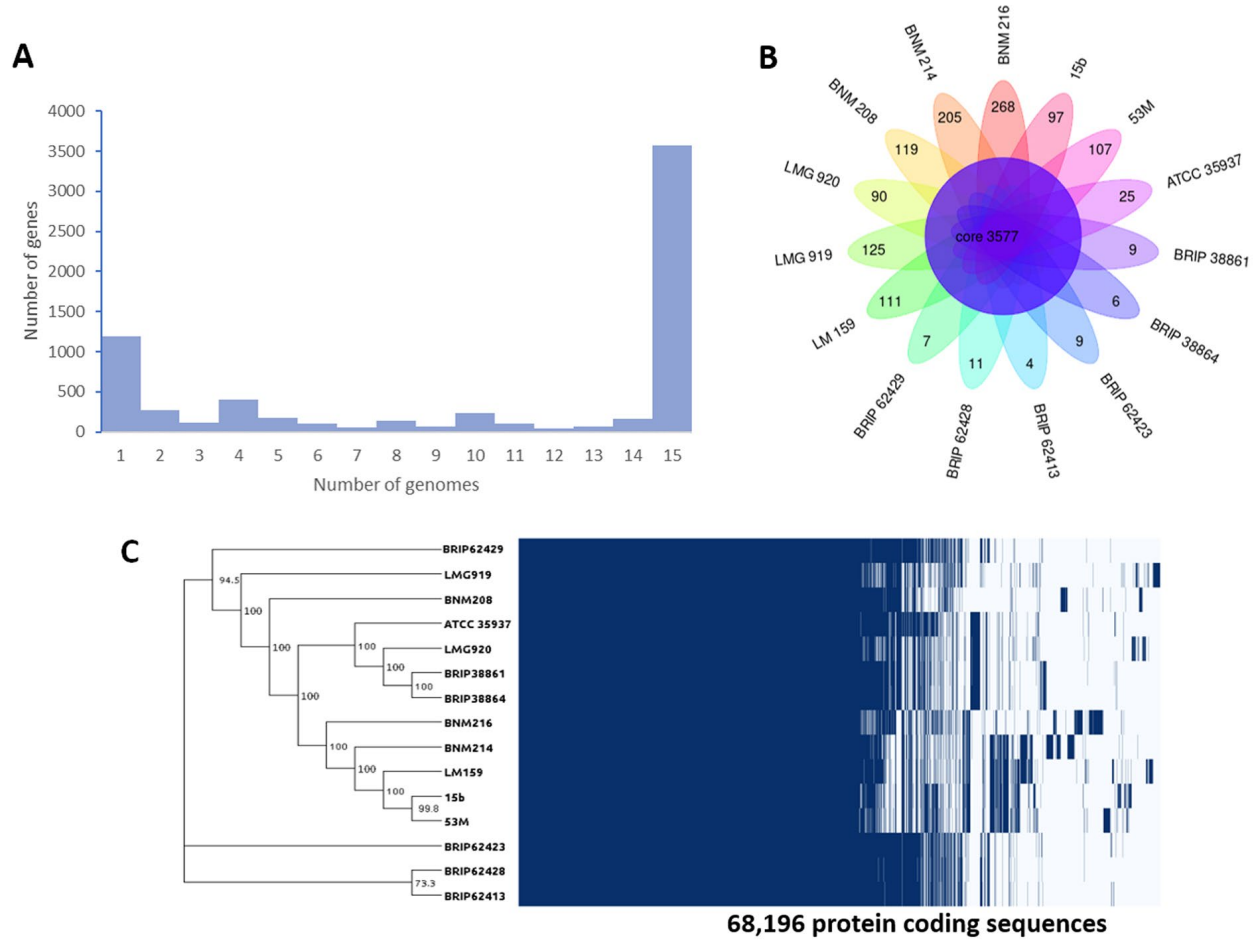
Genome comparative analysis between of *X. vesicatoria* strains BNM 208, BNM 214, BNM 216, and 12 *X. vesicatoria* strains. **(A)** Distribution of the 6,724 unique gene clusters per genome sequence. **(B)** Representation of genes shared among the 15 *X. vesicatoria* strains analysed in this work as well as BNM 208, BNM 214, and BNM 216 unique CDS. **(C)** Phylogenetic analysis of 15 strains of *X. vesicatoria* based on the core genome sequence (3,777 CDSs) and distribution of the 68,196 CDSs found in the pangenome of *X. vesicatoria*. Bootstrap values showed at the branch points.

When comparing the genome of BNM 208, BNM 214, and BNM 216 with those of 12 other representative *X. vesicatoria* strains (Supplementary Table S1), the average nucleotide identity (ANI) was more than 99% (Figure 1). This confirms that the three strains are members of *X. vesicatoria*. This analysis also showed that 3,577 core genes are shared by all 15 *X. vesicatoria* strains, that 3,147 accessory genes were identified (Figures 2A,B) and that a total of 68,196 CDSs were identified in the pangenome of *X. vesicatoria* (Figure 2C). BNM 208, BNM 214, and BNM 216 contained 119, 205, and 268 unique CDSs, respectively (Figure 2B).

#### 3.1.2. Gene ontology

According to the functional analysis based on the Clusters of Orthologous Genes (COGs), the CDSs were similarly distributed in the genomes of the three strains, and 25 out of the 26 COG functional categories were represented (Table 2).

**Table 2:** CDSs distribution in COG functional categories for the three *X. vesicatoria* genomes sequenced.

Description		Strains					
		BNM208		BNM214		BNM216	
		Value^a^	%	Value	%	Value	%
A	RNA processing and modification	2	0.01	2	0.01	2	0.01
B	Chromatin structure and dynamics	6	0.04	6	0.04	6	0.04
C	Energy production and conversion	960	6.10	876	5.72	881	5.78
D	Cell cycle control, cell division, chromosome partitioning	156	0.99	160	1.04	155	1.02
E	Amino acid transport and metabolism	1,800	11.44	1760	11.48	1,750	11.47
F	Nucleotide transport and metabolism	369	2.34	337	2.20	349	2.29
G	Carbohydrate transport and metabolism	879	5.58	860	5.61	848	5.56
H	Coenzyme transport and metabolism	425	2.70	417	2.72	414	2.71
I	Lipid transport and metabolism	345	2.19	331	2.16	329	2.16
J	Translation, ribosomal structure, and biogenesis	1,046	6.65	956	6.24	951	6.24
K	Transcription	1,122	7.13	1,134	7.40	1,108	7.27
L	Replication, recombination and repair	434	2.76	447	2.92	445	2.92
M	Cell wall, membrane, envelope biogenesis	528	3.35	513	3.35	527	3.46
N	Cell motility	535	3.40	521	3.40	521	3.42
O	Post-translational modification, protein turnover, chaperones	733	4.66	707	4.61	695	4.56
P	Inorganic ion transport and metabolism	1,505	9.56	1,469	9.59	1,471	9.65
Q	Secondary metabolites biosynthesis, transport and catabolism	196	1.25	194	1.27	190	1.25
R	General function prediction only	2,188	13.90	2,145	14.00	2,153	14.12
S	Function unknown	231	1.47	238	1.55	225	1.48
T	Signal transduction mechanisms	1,598	10.15	1,576	10.28	1,562	10.24
U	Intracellular trafficking, secretion, and vesicular transport	133	0.85	118	0.77	117	0.77
V	Defense mechanisms	487	3.09	484	3.16	487	3.19
W	Extracellular structures	35	0.22	31	0.20	31	0.20
X	Mobilome: prophages, transposons	24	0.15	42	0.27	32	0.21
Y	Nuclear structure	0	0.00	0	0.00	0	0.00
Z	Cytoskeleton	2	0.01	2	0.01	2	0.01

a Values based on the total number of CDS in the annotated genome. Each CDS could be assigned to more than one COG category.

**Figure 3:**
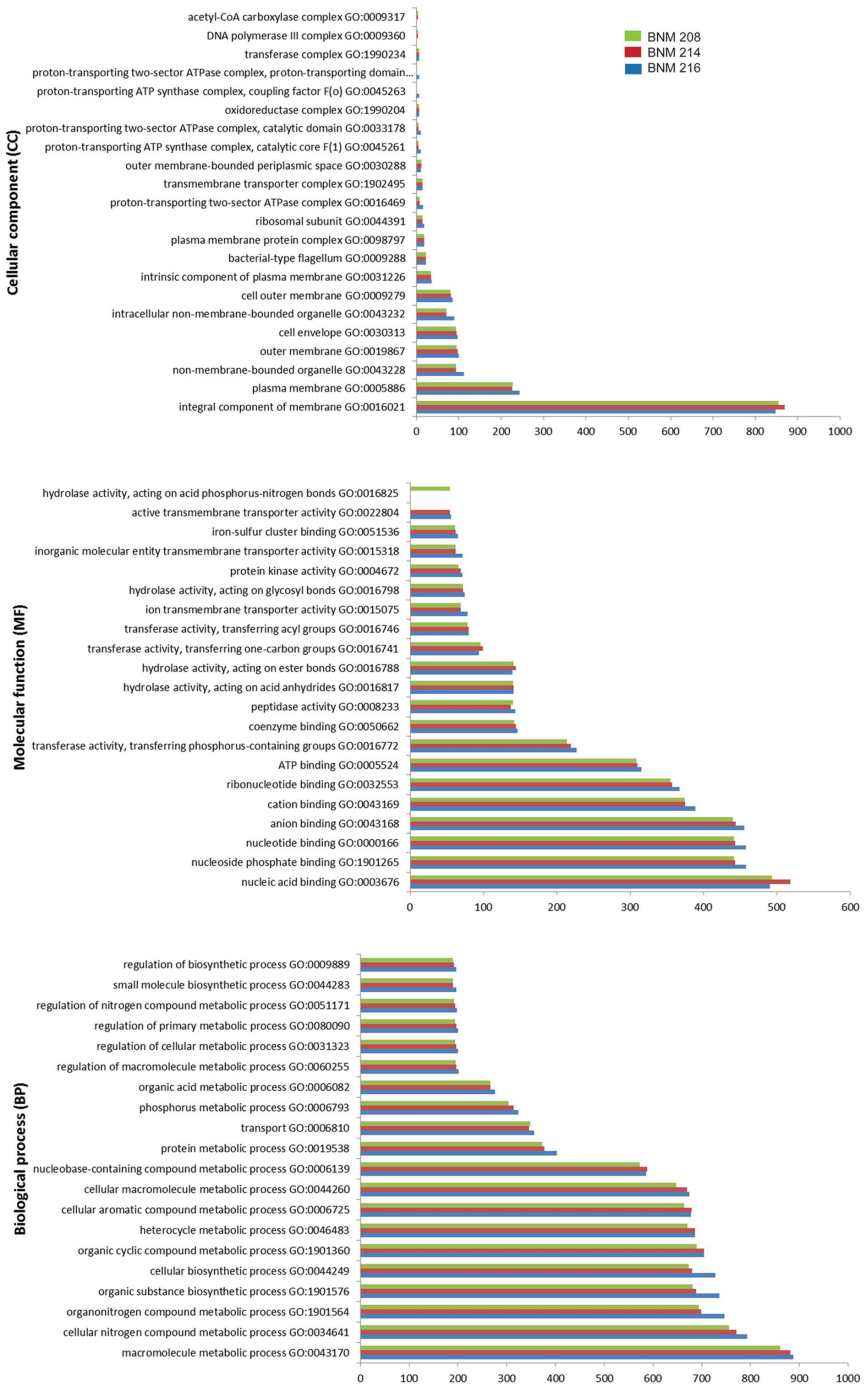
Number of gene coding sequences of *X. vesicatoria* strains BNM 208, BNM 214, and BNM 216 associated with the top 20 functions according to their cellular components (top panel), molecular functions (middle panel) and biological processes (bottom panel) in the GO database.

A general functional analysis was performed using the Gene Ontology (GO) database. Out of 4,454 potential gene sequences in BNM 208, 4,400 were associated with 967 molecular functions (MFs); 3,413 with 651 biological processes (BPs), and 1,777 with 75 cellular components (CCs) (Figure 3). Out of 4,671 potential gene sequences in BNM 214, 4,316 were assigned to 970 MFs, 3,358 to 650 BPs, and 1,742 to 75 CCs. Finally, out of 4,569 potential gene sequences in BNM 216, 4,265 were associated with 972 MFs, 3,333 with 648 BPs and 1,724 with 75 CCs. The leading 20 MFs, BPs and CCs for each strain are shown in Figure 3. The most abundant BPs were the macromolecule metabolic process (GO:0043170), the cellular nitrogen compound metabolic process (GO:0034641), and the organonitrogen compound metabolic process (GO:1901564). In the case of MFs, the most abundant were nucleic acid binding (GO:0003676), nucleoside phosphate binding (GO:1901265), and the nucleotide binding function (GO:0000166). The most abundant CCs comprised the integral components of the membrane (GO:0016021) and plasma membrane (GO:0005886), as well as components of the non-membrane bound organelles (GO:0043228).

#### 3.1.3. Genomic analysis of factors involved in virulence

The presence of more than 400 genes known to be functionally associated with pathogenesis in *Xanthomonas*, was determined in the genomes of the studied strains. As a whole, they showed a very similar repertoire. CDSs related to virulence enzymes were the same in BNM 208, BNM 214, and BNM 216. They consisted of at least 19 cellulolytic enzymes, 13 hemicellulolytic enzymes, 12 pectolytic enzymes and two lipases (Supplementary Table S3). Moreover, the strains had the same pattern for potential CDSs associated with chemotaxis, quorum sensing, flagellum components and regulators, xanthan gum production, and the components of secretion systems types II (T2SS) and III (T3SS) (Supplementary Table S3).

Slight variations were found in those genes that code for elements involved in sensing environmental changes, such as methyl-accepting chemotaxis proteins (MCPs), sensors of the two-component regulatory system (STCRs) and TonB-dependent transporters (TBDTs). Although the pattern of 23 MCPs-related genes was similar in the three strains, a CDS homologous to XCV1778 (NCBI RefSeq ID: WP_029819220) was absent in BM216. Out of the 70 genes that encode STCRs, xac0610 homologous (AAM35499.1) and *xcv3267* (CAJ24998.1) were not present in BNM 216 and BNM 214, respectively. Finally, out of the 26 TBDT-encoding genes, only BNM 216 lacked sequenced homologous to *xcc1340* (AAM40638.1) (Supplementary Table S3).

#### 3.1.4. Pilin related *in-silico* analyses

**Figure 4:**
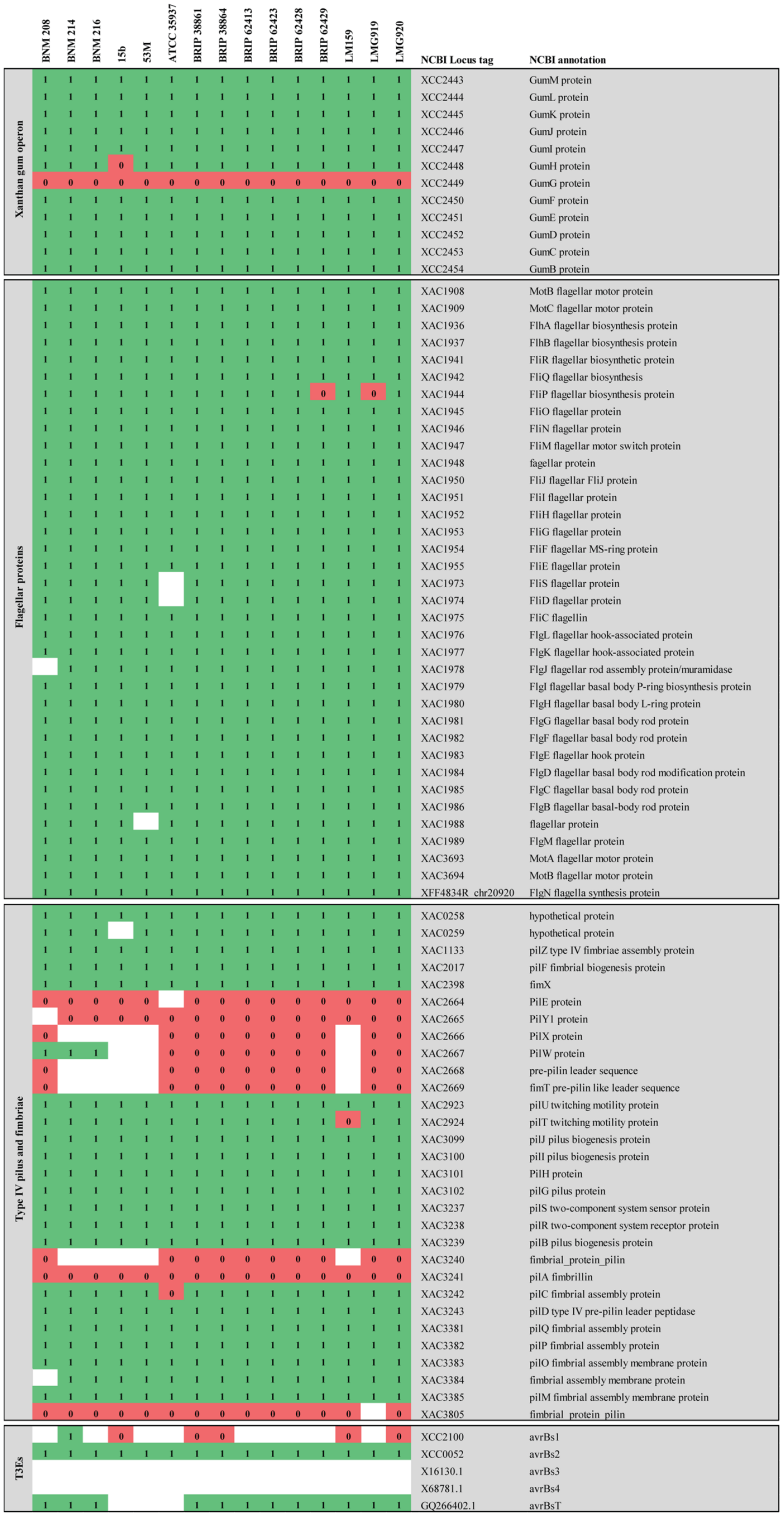
Comparative genomic analysis of CDSs associated with virulence-related genes described in *Xanthomonas*. The comparative analysis included strains BNM208, BNM214, BNM216, and 12 other *X. vesicatoria* strains whose genomes are available in databases. This figure shows only the results of the FIGURE 4 (Continued)analysis for clusters associated with xanthan, flagellum and type IV pili (T4P) and for genes encoding six effector proteins secreted by the type III secretion system (TE3s) associated with host specificity and genes encoding sensors of the two-component regulatory system (STCRs). Comparative genomic analysis for other genes associated with T3Es and STCRs is shown in the Supplementary Table S3. Each of the genes analysed is represented by its locus tag number at the National Center for Biotechnology Information (NCBI) database. Xac, *X. citri* subsp. *citri*; Xcc, *X. campestris* pv. *campestris*; Xff, *Xanthomonas fuscans* subsp. *fuscans*. The similarity/identity of homologous sequences showing a percentage higher than 80% (1) are shown in green, those showing a percentage lower than 80% (0) are shown in red and homologous sequences not found are shown in white.

**Figure 5:**
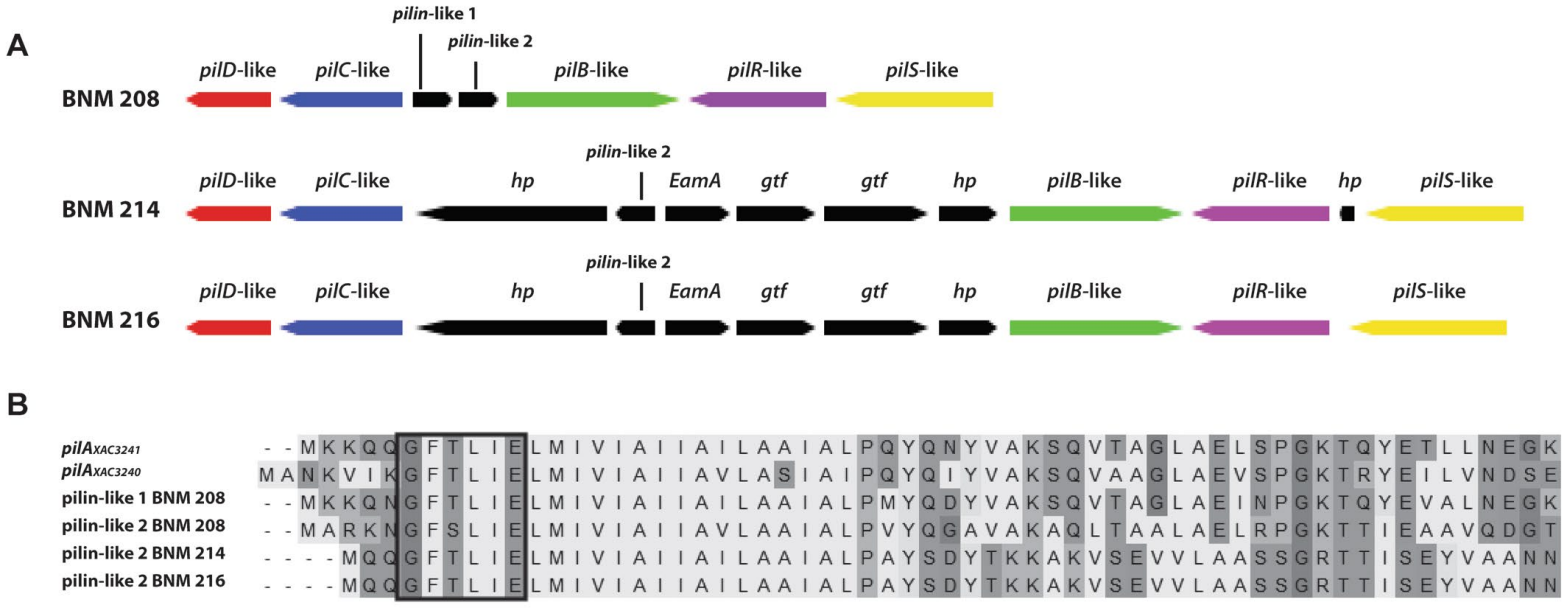
*In silico* analysis of some genes involved in the biosynthesis of T4P. **(A)** Schematic representation of the set of genes *pilSRBCD* found in the BNM 208, BNM 214, and BNM 216 genomes (*hp*, hypothetical protein; *gtf*, glycosyltransferase; *EamA*, family transporter RarD). **(B)** Amino acid alignment of proteins encoded by *pilin-*like 1 and *pilin*-like 2 genes of BNM 208, BNM 214, and BNM 216 with *pilA_XAC3241_* and *pilA_XAC3240_* of Xac306. The prepilin leader sequence (signal peptide) is marked with a box. The alignment was carried out using ClustalW.

The *pilSRBACD* gene cluster (Dunger et al., 2014), which encodes for major pilin PilA and minor pilins, was present in the three strains with some differences. Sequences homologous to *pilS _XAC3237_*, *pilR _XAC3238_, pilB _XAC3239_ pilC _XAC3242_ and pilD _XAC3243_* in Xac306 (hereafter referred to as *pilSRBCD*) were found in our three *X. vesicatoria* strains (Supplementary Table S3 and Figure 4). In BNM 208, two genes (*pilin*-like 1 and *pilin*-like 2), were detected between *pilB-*like and *pilC-*like genes. The two were homologous to the fimbrillins *pilA_Xac3241_* (AAM38085.1) and *pilA_Xac3240_* (AAM38084.1) in Xac306, though the similarity/identity percentage was under 80% (Supplementary Table S3 and Figure 4). In BNM 214 and BNM 216, six genes were detected between *pilB-*like and *pilC*-like genes. The first encodes a hypothetical protein (*hp*), the second one is *pilin-*like 2, the third codes for a family transporter RarD (*EamA*), the next two for glycosyltransferases (*gtf*), and the last one for another hypothetical protein (Figure 5A). The amino acid sequences of the Pilin-like proteins in our strains were aligned for comparison with those of homologous major pilins in Xac306 (PilA_XAC3240_ and PilA_XAC3241_). Both Pilin-like 1 and Pilin-like 2 contain a G-1F + 1/E+5 motif, that is characteristic of type IVa pilins (Figure 5B). Besides, the *in-silico* analysis of the tertiary structures of Pilin-like 1 and Pilin-like 2 showed that their predicted structures are similar to PilA_XAC3241_ and PilA_XAC3240_ of Xac306 (Supplementary Figure S1).

In terms of minor pilins involved in virulence in other species, the three strains in this study had a similar sequence to *xac3805* (AAM38647.1) in Xac306 (named *DUF4339 domain containing protein* gene, henceforth referred to as *DUF4339*). They also presented a CDS homologous to *pilE_Xac2664_* (AAM37511.1). On the other hand, a CDS homologous to *pilY1_Xac2665_* was detected in BNM 214 and BNM 216 although with less than 80% similarity/identity (Supplementary Table S3 and Figure 4). Potential CDSs homologous to *pilX_xac2666_* (AAM37513.1) and *fimT_xac2669_* (AAM37516.1) in Xac306 were absent in BNM 214 and BNM 216. However, BNM 208 did harbour potentially homologous sequences to these genes, although the similarity/identity percentage was once again under 80% (Supplementary Table S3 and Figure 4).

#### 3.1.5. *In-silico* analyses for T3Es

Differences between the three strains were also noted in the repertoire of potentially homologous T3E-encoding sequences. Out of 38 CDSs associated with these effectors, 26 were found in BNM 208, the most virulent strain (10 with a similarity/identity percentage over 80% and 16 with a similarity/identity percentage under 80%), 29 in BNM 214 (13 with a similarity/identity percentage over 80% and 16 with a similarity/identity percentage under 80%), and 25 in the least virulent strain, BNM 216 (8 with a similarity/identity percentage over 80% and 17 with a similarity/identity percentage under 80%). Supplementary Table S3 shows the presence/absence of each of these 38 T3E-asociated CDs in the 15 genomes analysed, with the main differences pertaining to our strains mentioned below. Potentially homologous sequences to *xopR* (KT873949.1), *xopD* (CAJ22068.1), *xopE4* (EFF46466.1), *xopAG* (EGD09323.1), *xac0277* (AAM35169), *xac0543* (AAM35432.1) and *xcc3600* (AAM42870.1) were not detected in any of our three strains (Supplementary Table S3).

Only BNM 214 has a complete sequence of *avrBs1* (AAM41388.1) with over 80% similarity to that in Xac306. In the three strains, analysis of amino acid sequences corresponding to *avrBs3* (CAA34257.1) and *avrBs4* (CAA48680.1) showed a partial nucleotide sequence that mapped about 2,000 bp out of a total of 4,000 bp for the described genes. There were no potentially homologous sequence to *xopE2* (AAM39257.1), *xopH1* (CAJ19917.1), or *xopE3* (AAM38068.1) in BNM 216, or to *xopN* (AAM37631.1) in BNM 214 and BNM 216. Similarly, potentially homologous sequences to *xopX* (EGD08143.1) and *xac0543* (AAM35432.1) were undetected in BNM 208 (Supplementary Table S3).

### 3.2. *In vitro* analysis of virulence factors that contribute to biofilm formation and/or virulence

#### 3.2.1. Fimbrial adhesins involved in bacterial adhesion and motility: flagella and T4P

**Figure 6:**
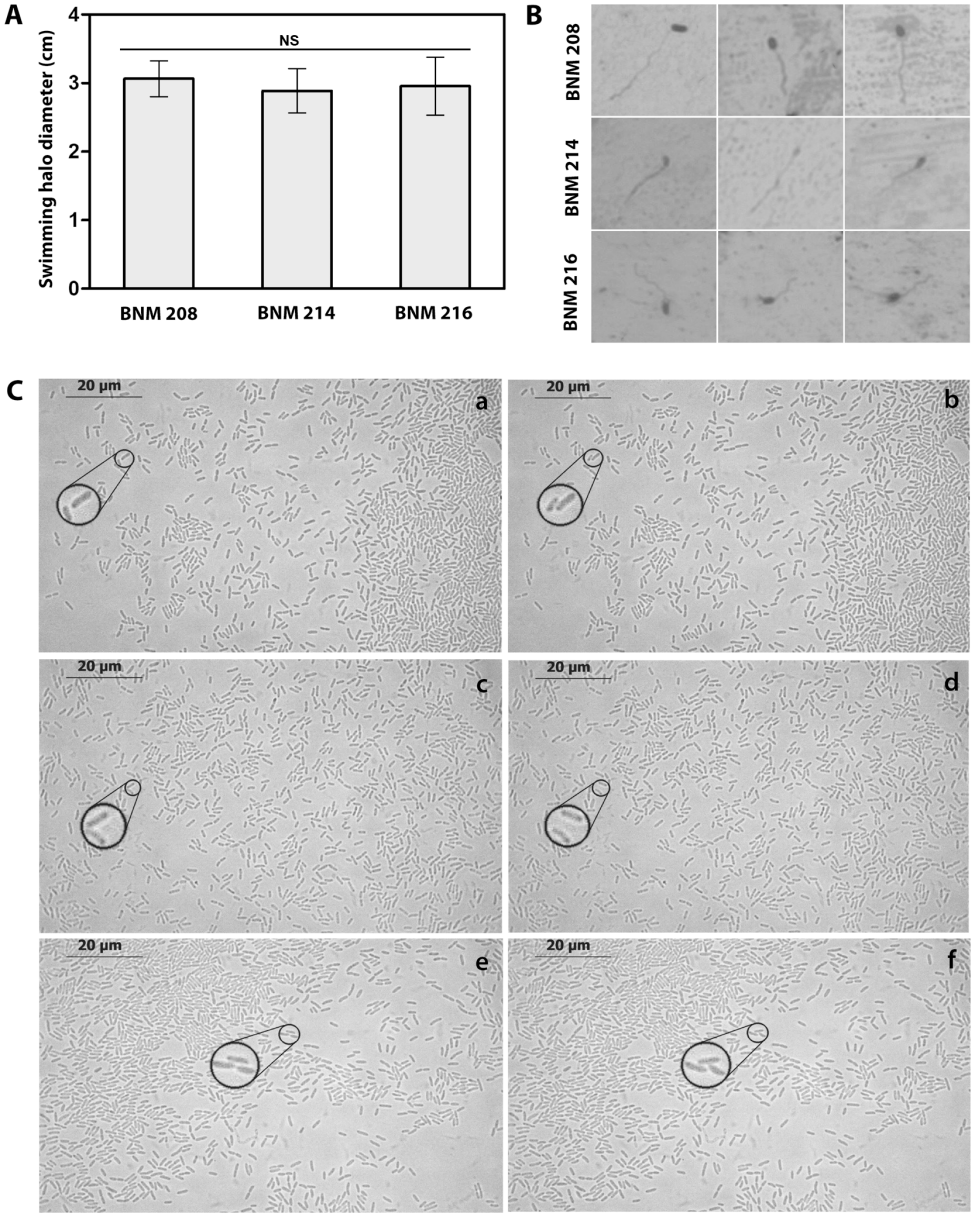
Functional analysis of flagella and T4P. **(A)** Swimming halo diameter of BNM 208, BNM 214, and BNM 216 cultured in PYM agar. Letters “NS” means no significant difference according to one-way ANOVA followed by Tukey’s test at a significance level of *p* ≤ 0.05. **(B)** Microscopic observation (1,000×) of the single polar flagellum of BNM 208, BNM 214, and BNM 216. **(C)** Twitching motility of BNM 208 (panels a and b), BNM 214 (panels c and d) and BNM 216 (panels e and f) analysed by optical microscopy (1,000×) by time-lapse observations. The right panels (b, d, and f) show images taken 30 s later than the images from the left panels (a, c and e). To easily visualize the typical twitching motility zoom was placed in regions of the observed fields, for each strain, in the reported time lapse.

Previously, we reported significant differences in swarming (flagellum-dependent movement) between BNM 208, BNM 214, and BNM 216 (Felipe et al., 2018). Considering that swarming also depends on other factors than flagella, in this work, we studied swimming motility, which is mainly dependent on the flagella. Our results showed that the three strains were able to swim at similar levels (Figure 6A). In addition, we observed by optical microscopy a single polar flagellum in BNM 208, BNM 214, and BNM 216 cells (Figure 6B).

Twitching motility, which depends on T4P was observed by time-lapse microscopy as characteristic sudden movements by the individual cells of all three strains. Changes in position corresponding to this bacterial movement are shown in Figure 6C and Supplementary Figure 1.

#### 3.2.2. Xanthan

**Figure 7:**
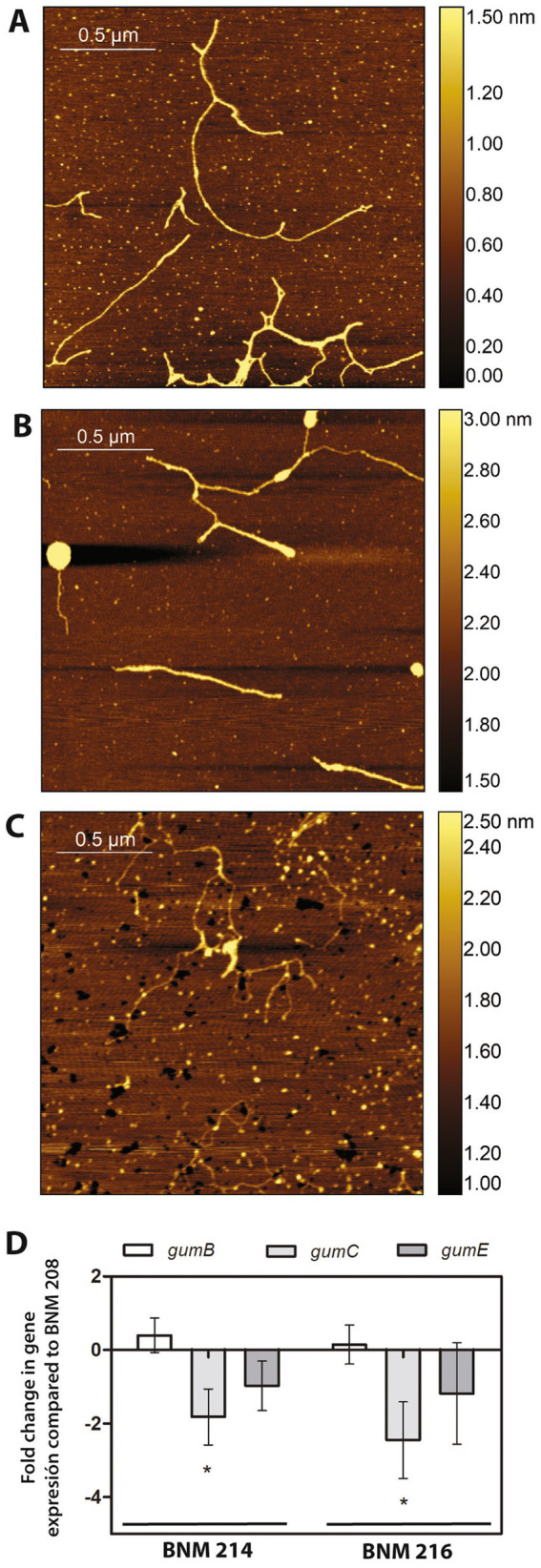
Xanthan analysis. **(A–C)** Chains of xanthans produced by BNM 208, BNM 214, and BNM 216, respectively. Representative AFM images were obtained from over 200 observed chains/strands. **(D)** Expression of *gumB*, *gumC*, and *gumE* in BNM 214 and BNM 216 relativised to the respective genes in BNM 208.

As mentioned in Section 3.1.3, the genomes of BNM 208, BNM 214, and BNM 216 were no different in their *gum* genes. However, in previous work, we reported that xanthans produced by these strains showed significant different viscosities (Felipe et al., 2018). Since the chain-length of xanthan molecules has been reported to affect xanthan viscosity (Galvan et al., 2013), the EPS molecules synthesised by the three strains were analysed by AFM. According to the images, BNM 208 produced longer xanthan molecules than the other two strains. The shortest xanthan molecules were produced by BNM 216 (Figures 7A–C).

Next, qPCR was performed to explore the expression of *gumB*, *gumC* and *gumE* genes, which are involved in xanthan polymerization and export. These genes are related to xanthan chain-length (Galvan et al., 2013). The expression of *gumC* was approximately 2 and 2.5 times less in BNM 214 and BNM 216 than in BNM 208, respectively. No significant differences were registered in the expression levels of *gumB* or *gumE* between the three strains (Figure 7D).

### 3.3. *In planta* analysis to verify the host specificity in tomato

Differential tomato genotypes are used to identify races based on HR expression (Jones et al., 2004). The three differential lines used in this study (*S. lycopersicum* lines Bonny Best and Hawaii 7998 and *S. pimpellifolium* PI 128216) resulted in susceptible reactions following inoculation with BNM 208, BNM 214, and BNM 216, indicating the three strains belong to race T2 (data not shown).

## 4. Discussion

*X. vesicatoria* and *X. euvesicatoria* were historically considered the predominant bacterial spot lineages worldwide, although *X. perforans* and *X. gardneri* are increasingly isolated from areas in America, Africa, the Middle East, and the Indian Ocean (Vancheva et al., 2021). However, the disease caused by *X. vesicatoria* continues to be a worldwide problem due to the seedborne nature of this pathogen (Kebede et al., 2014; Potnis et al., 2015; Berrueta et al., 2016; Roach et al., 2018; Osdaghi et al., 2021; Potnis, 2021; Ahmad and Ahmad, 2022).

This study aimed to elucidate the factors that could be associated with the development of bacterial spot on tomato plants caused by *X. vesicatoria*. To achieve this goal, we analysed three strains of *X. vesicatoria*, BNM 208, BNM 214, and BNM 216, which had shown significant differences in aggressiveness in a previous study (Felipe et al., 2018). The data collected at that time suggested that those differences might be connected to variations in the ability of these strains to form biofilm, which in turn may respond (at least in part) to differences in bacterial motility and in the rheological properties of the xanthan produced by each strain. The most virulent strain, BNM 208, was able to form a mature, homogeneous and well-structured biofilm, while the least virulent one, BNM 216, formed a biofilm that was practically detached and had no characteristics indicating maturity (Felipe et al., 2018).

Since biofilm formation is a complex process involving numerous microbial components and produced/secreted compounds, such as EPSs (An et al., 2020), here, we studied some microbial features associated with aggregation mechanisms. Little is known about the processes that allow *X. vesicatoria* to survive, multiply and establish itself in its hosts. The same can be said for virulence factors related to host colonization or the evasion of the host’s defense response. Understanding which of these factors are key in pathogenesis and how their repertoire varies from one strain to another could go a long way in explaining differences in aggressiveness. For this reason, we compared the genomes of BNM 208, BNM 214, BNM 216, not only with each other but also with those of 12 other *X. vesicatoria* strains, which are available in public databases (Supplementary Table S1). Our three strains were confirmed to be *X. vesicatoria*. Despite their different degrees of aggressiveness and phenotypic characteristics (e.g., motility, adhesion, biofilm-forming ability, relative viscosity of xanthan), their genomes had similar functional patterns in terms of GO and COG categories (Figure 3 and Table 2).

An important virulence factor related to biofilm formation is xanthan. BNM 208, BNM 214 and, BNM 216 contain all the potential CDSs encoding gum proteins, which are involved in the synthesis of xanthan repetitive units, its decoration with non-glycosidic groups (except *gumG*), polymerization, translocation to the periplasm, and export to the extracellular medium. Therefore, the differences in the viscosity of the xanthans synthesised by these strains (Felipe et al., 2018) cannot be attributed to differences in *gum* genes was. However, a molecular chain-length analysis showed that the xanthan strands produced by BNM 208 are the longest, while those produced by BNM 216 are the shortest (Figure 7). Moreover, the g*umC* gene was expressed significantly more in BNM 208 than in the other two strains. These results are consistent with those reported by Galvan et al. (2013), according to whom xanthan with longer molecules results in a more viscous EPS than xanthan with shorter ones.

Xanthan is also important as a surfactant for swarming motility: its amphipathic characteristics reduce the tension between the substrate and the bacterial cells, and thus enhance their spread on solid surfaces (Kearns, 2010). Since we did not observe differences in the CDSs encoding flagellum components (Supplementary Table S3), we cannot attribute the differences observed earlier in swarming motility between BNM 208, BNM 214, and BNM 216 (Felipe et al., 2018) to genetic differences between the three strains. Furthermore, we observed by light microscopy that all three strains have a unique flagellum, the functionality of which was verified by swimming assays (Figures 6A,B). Their differing ability to swarm might be due to the characteristics of the xanthans produced by BNM 208, BNM 214, and BNM 216.

T4P is another factor that influences virulence, the initial stages of biofilm formation and maturation and other bacterial functions in many bacteria species (Jarrell and McBride, 2008; Dunger et al., 2016; Dubnau and Blokesch, 2019; Ellison et al., 2022). Its role in the pathogenesis of *Xanthomonas* is not the same across all species (Dunger et al., 2016; Shah et al., 2021). The gene(s) that encode(s) PilA remain unknown in *X. vesicatoria*. Two genes named *pilA_XAC3241_* and *pilA_XAC3240_* have been reported to code for this protein in Xac306 (Dunger et al., 2014). The presence/absence of these genes in the genomes of our strains was studied by genomic and PCR analyses. Homologs of *pilA_XAC3240_* were not found in any of the three strains, and a homologous sequence to *pilA_XAC3241_* was only found in BNM 208 (Supplementary Table S4).

The ability of BNM 208, BNM 214, and BNM 216 to move by twitching was assessed as well, since it depends exclusively on the presence of a functional T4P. Instead of examining the edges of the colonies formed by each strain, as in our previous work (Felipe et al., 2018), we now tracked the movement of individual bacterial cells at very short time intervals (Figure 6C). This technique revealed that BNM 208, BNM 214, and BNM 216 are all capable to move by twitching, whereas earlier results (Felipe et al., 2018) shown this type of movement only in BNM 208 and BNM 214, probably due to the technique used in that previous work had not allowed us to observe movement of individual bacterial cells.

We delve into the analysis of the cluster where the gene(s) encoding *pilA* should be found according to the literature (Dunger et al., 2014). As in most *Xanthomonas* genomes, in our strains the genes that code for major pilins are part of a cluster that also contains *pilS, pilR, pilB, pilC and pilD.* According to Dunger et al. (2014), *pilA_XAC3241_* and *pilA_XAC3240_* are located between *pilB* and *pilC* in the *pilSRBACD* gene cluster. In BNM 208, BNM 214, and BNM 216, we also found a *pilSRBCD* cluster with some differences between the three strains. BNM 208 has two genes between the *pilB-*like and *pilC-*like genes, named *pilin-*like 1 and *pilin-*like 2, which might, respectively, correspond to *pilA_XAC3241_* and *pilA_XAC3240_*. In contrast, BNM 214 and BNM 216 have six genes between the *pilB-*like and *pilC-*like genes, including *pilin-*like 2. A protein alignment and structure analysis revealed that Pilin-like 1 and Pilin-like 2 proteins in the three strains contain the motif G-1F + 1/E+5 (Figure 5B), and that their predicted tertiary structures are similar to those of PilA_XAC3241_ and PilA_XAC3240_ in Xac306. These results could serve as the basis for more accurate identification and functional characterisation of T4P pilins in *X. vesicatoria*.

The *DUF4339* gene, which is homologous to *xac3805* of Xac306, is present in the genomes of BNM 208, BNM 214, and BNM 216. Its interest lies in the fact that *xac3805* encodes a pilin-like protein that was initially annotated as *pilA* (da Silva et al., 2002). However, Dunger et al. (2014) later noted that it did not feature the characteristic prepilin leader sequence of type IVa pilins, and it is located far from the *pilSRBACD* genes. Since the same observations were made for *DUF4339* in our *X. vesicatoria* strains, it might actually be a pilin-encoding paralogue of *pilA*. Still, further studies should determine whether it encodes a minor or major pilin, and the significance of that pilin for T4P functionality.

Functional studies with Xac306 mutants defective in PilA, PilB, PilZ, or FimX showed that they were impaired in their ability to adhere to the leaf surface, which highlights the importance of these proteins for the formation of stable biofilms (Dunger et al., 2014). Likewise, the involvement of PilA in bacterial adhesion was demonstrated with PilA-defective mutants of *Xanthomonas fuscans* subsp. *fuscans* (Darsonval et al., 2009). Other examples are FimA mutants of *X. campestris* pv. *vesicatoria*, which exhibited dramatically reduced cell aggregation *in vitro* and on tomato leaves, and decreased tolerance to ultraviolet light (Ojanen-Reuhs et al., 1997). In BNM 214 and BNM 216, the absence of minor pilin-encoding genes homologous to *pilE_Xac2664_*, *pilX_xac2666_* and *fimT_xac2669_*, and *pilA_xac3241_* (which was confirmed by PCR analysis; see Supplementary Tables S3, S4) could explain why the biofilms formed by these two strains are not as mature and well-structured as the one developed by BNM 208.

There are a few differences in the genomes of the three strains as far as STCRs are concerned. BNM 208 and BNM 214 have a sequence homologous to *xac0610* (AAM35499.1). In Xac306, this gene encodes a protein with diguanylate cyclase (DGC) activity, associated with the presence of a GGDEF domain that leads to the production of cyclic diguanosine monophosphate (c-di-GMP) (Oliveira et al., 2015). In addition, the three strains have a sequence homologous to *xac1345* (AAM36216.1), which encodes another protein with a GGDEF domain in Xac306 (Andrade et al., 2006). However, its similarity/identity percentage was under 80% in BNM 216 (Supplementary Table S3 and Figure 4). Cyclic-di-GMP is a ubiquitous second messenger in bacteria and a signaling molecule whose intracellular levels are decisive in the transition from a planktonic to a sessile lifestyle that lead to biofilm formation, among other functions (Valentini and Filloux, 2019). In many bacteria, an increase in c-di-GMP levels raises the expression of several factors necessary for the establishment and maintenance of biofilm. Our genomic findings on *xac0610* and *xac1345* (Supplementary Table S3 and Figure 4) were verified by PCR. The sequence homologous to *xac0610* was only amplified in BNM 208, while that homologous to *xac1345* was only amplified in BNM 208 and BNM 214. Again, more in-depth research should elucidate the role of these genes in biofilm formation by *X. vesicatoria*.

On the other hand, since T3Es are associated with host specificity and pathogenicity and play an essential role in the early steps of bacterial interaction with host and non-host plants (Alavi et al., 2008), we analysed the repertoire of CDSs encoding them in BNM 208, BNM 214, and BNM 216 and found several differences between the three strains (Supplementary Table S3 and Figure 4).

Finally, since BNM 208, BNM 214, and BNM 216 are *X. vesicatoria* and caused symptoms in *S. lycopersicum* lines Bonny Best and Hawaii 7,998 and in *S. pimpellifolium* PI 128216, we concluded that all three strains belong to race T2.

The novel findings presented in this paper contribute to our comprehension of the mechanisms underlying biofilm formation and pathogenicity in *X. vesicatoria*. To our knowledge, this is the first report that studied genotype and phenotype parameters of key virulence factors in this species, including xanthan, flagella and T4P. In conclusion, this study provides information on the biology and pathogenicity of *X. vesicatoria,* which is still scarce, and contributes to the knowledge of this important plant pathogen. While this work represents a crucial starting point, deeper analyses, such as the construction and examination of mutant strains in various virulence factors or transcriptomic analyses, are needed to further elucidate the underlying mechanisms involved. The final goal of our study is to use this knowledge for designing disease control tools addressed to interfere or attenuate bacterial pathogenicity mitigating the devastating impact of *X. vesicatoria* infections on crops.

## Data availability statement

The datasets presented in this study can be found in online repositories. The names of the repository/repositories and accession number(s) can be found below: https://www.ncbi.nlm.nih.gov/genbank/, PRJNA610021 (BNM 208), PRJNA610030 (BNM 214) and PRJNA610032 (BNM 216).

## Author contributions

MB: conceptualisation, research and formal analysis, writing of the original draft, and revision and editing of the manuscript. MP: research and formal analysis, preparation and editing of some figures, contribution to the writing of the original draft, and revision of the final manuscript. JG-C: assembly and analysis of *X. vesicatoria* genomes, preparation and editing of some figures, contribution to the writing of the original draft, and revision of the final manuscript. VC: gene expression assays. TG: contribution to the analysis of *X. vesicatoria* genomes and revision of the original and final draft. GD: methodological and analytical support for some experiments and revision of the original and final draft. GM: AFM tests, formal analysis of the images, and review of the final manuscript. AV: research support and review of original and final drafts. AR: trials on tomato plants and revision of the original and final draft. JC: conceptualisation support and assembly and analysis of *X. vesicatoria* genomes, contribution to the writing of the original draft, and revision of the final manuscript. PY: fund acquisition and project management, conceptualisation, research supervision and formal analysis, and contribution to the correction of the original draft and revision of the final manuscript. All authors contributed to the article and approved the submitted version.

## Funding

This work was funding by the Fondo para la Investigación Científica y Tecnológica (FONCyT) of the Agencia Nacional de Promoción de la Investigación, el Desarrollo Tecnológico y la Innovación (Argentina): PICT 2019 N° 3274.

## Conflict of interest

The authors declare that the research was conducted in the absence of any commercial or financial relationships that could be construed as a potential conflict of interest.

## Publisher’s note

All claims expressed in this article are solely those of the authors and do not necessarily represent those of their affiliated organizations, or those of the publisher, the editors and the reviewers. Any product that may be evaluated in this article, or claim that may be made by its manufacturer, is not guaranteed or endorsed by the publisher.

## Footnotes

^1^ http://rsbweb.nih.gov/ij/download.html

^2^ http://gwyddion.net/
