# Supplementary material for: Genomic and phenotypic insight into Xanthomonas vesicatoria strains with different aggressiveness on tomato
Source: Front Microbiol. 2023 Jun 27;14:1185368. doi: 10.3389/fmicb.2023.1185368 (PMC10333488; doi:10.3389/fmicb.2023.1185368)
Supplement: Supplementary file 1 [file Data_Sheet_1.docx]

Supplementary Material

**Genomic and phenotypic insight into *Xanthomonas vesicatoria* strains with different aggressiveness on tomato**

**María Isabel Bianco^*^, María Agustina Ponso, Jerson Garita-Cambronero, Valeria Paola Conforte, Tadeo E. Galván, Germán Dunger, Gustavo M. Morales, Adrián Alberto Vojnov, Ana María Romero, Jaime Cubero, Pablo Marcelo Yaryura^*^**

*** Correspondence:** Corresponding Author: pyaryura@unvm.edu.ar, ibianco@centromilstein.org.ar

**Supplementary Table S1:** *Xanthomonas vesicatoria* strains used in the comparative genomic study

| **Strain** | **NCBI**  **genome accession** | | **Origin** | | **Host** | | **Reference** | |
| --- | --- | --- | --- | --- | --- | --- | --- | --- |
| **BNM208** | PRJNA610021 | Argentina | | Hybrid Gaucho tomato | | Felipe et al., 2018 | |  |
| **BNM214** | PRJNA610030 | Argentina | | Hybrid FSO tomato | | Felipe et al., 2018 | |  |
| **BNM216** | PRJNA610032 | Argentina | | Hybrid Llanero tomato | | Felipe et al., 2018 | |  |
| **15b** | JSXZ00000000 | Bulgaria | | Pepper | | Vancheva et al., 2015 | |  |
| **53M** | JSYJ00000000 | Macedonia | | Pepper | | Vancheva et al., 2015 | |  |
| **ATCC 35937** | GCA_001908725.1 | New Zealand | | Tomato | | Potnis et al. 2011 | |  |
| **BRIP 38861** | QEYZ0100000 | Australia | | Tomato | | Roach et al. 2019 | |  |
| **BRIP 38864** | QEZA00000000 | Australia | | Peruvian Tomato | | Roach et al. 2019 | |  |
| **BRIP 62413** | QEZF00000000 | Australia | | Tomato | | Roach et al. 2019 | |  |
| **BRIP 62423** | QEZE00000000 | Australia | | Tomato | | Roach et al. 2019 | |  |
| **BRIP 62428** | QEZC00000000 | Australia | | Tomato | | Roach et al. 2019 | |  |
| **BRIP 62429** | QEZB00000000 | Australia | | Tomato | | Roach et al. 2019 | |  |
| **LM159** | GCA_001908815.1 | Argentina | | Pepper | | Richard et al. 2017 | |  |
| **LMG919** | JTEF00000000 | Zimbabwe | | Tomato | | Lefeuvre, 2014. Unpublished | |  |
| **LMG920** | JTEG00000000 | Italy | | Tomato | | Lefeuvre, 2014. Unpublished | |  |

**Supplementary Table S2.** Primers used for PCR and qPCR assays

|  | **Gene** | **Forward primer 5' - 3'** | **Reverse primer 5' - 3'** | **Amplicon (bp)** |
| --- | --- | --- | --- | --- |
| **PCR** | ***pilE_xac2664_*** | TAAATCCCGTCGTTCGGCTG | CGTTCTGCTTCGAGGTTGGA | 203 |
|  | ***pilX_xac2666_*** | GACTCTTGACCACCTACGGC | ACTCACAGCGGTAATGCGAT | 464 |
|  | ***pilW_xac2667_*** | GTGGTCGCAATGCCAAGG | GCGAGTTGTAACACGGAAGC | 634 |
|  | ***fimT_xac2669_*** | CGCGTGTTATTGCTACACGG | AAGTCCTGCGTGTCAGTGAG | 228 |
|  | ***pilA_xac3241_*** | ATCAATCCTGGCAAGACGCA | ACGCGTCAATGTCACCTTCT | 201 |
|  | ***pilA_xac3240_*** | CTGGTAGCAGTCGCAGTCAA | CTATAATCGCGGTGCTTGCT | 187 |
|  | ***Xac0610*** | TGGGGGTGCTGGACACCGAGGCCGA | GCCTACGGCACCTTGTGCACGCTC | 338 |
|  | ***Xac1345*** | GCAGAAGGACGGTGAATTCC | ATAACCGCCGCTATCTGGAA | 237 |
| **Q-PCR** | ***gumE*** | CGGCATATCAAGACACCAC | TCAGCAACAGGAACGAATAC | 198 |
|  | ***gumC*** | AGATCGCCAACACCTATCC | CTTTTCCGAGTCTTCGACC | 134 |
|  | ***gumB*** | GACCGAGATAGAGAAGGGTG | ACTAAGGGGGTCAGTTCCAG | 120 |

**Supplementary Table S3:** Comparative genomic analysis of CDSs associated with virulence-related genes described in *Xanthomonas*. The analysis included strains BNM 208, BNM 214, BNM 216, and 12 other *X. vesicatoria* strains whose genomes are available in databases. Each analysed gene is represented by its locus tag number at the National Center for Biotechnology Information (NCBI) database. XAC, *Xanthomonas citri* subsp. citri; XCC, *Xanthomonas campestris* pv. campestris; XC, *Xanthomonas campestris*; XCV, *Xanthomonas* *campestris* pv. *vesicatoria*; XCR, *Xanthomonas campestris* pv. *raphani*; XOO, *Xanthomonas oryzae* pv. *oryzae*; XOC, *Xanthomonas oryzae* pv.*oryzicola*; XFF, *Xanthomonas fuscans* subsp. fuscans. The similarity/identity of homologous sequences showing a percentage higher than 80 % (1) are shown in green, those showing a percentage lower than 80 % (0) are shown in red and homologous sequences not found are shown in white.

*continuation of Supplementary Table 3*

**

*continuation of Supplementary Table 3*

*continuation of Supplementary Table 3*

**

*continuation of Supplementary Table 3*

*continuation of Supplementary Table 3*

*continuation of Supplementary Table 3*

*continuation of Supplementary Table 3*

*continuation of Supplementary Table 3*

*continuation of Supplementary Table 3*

**Supplementary Figure S1**: Predicted tertiary structure of Pilin-like 1 and Pilin-like 2 proteins of BNM 208 and Pilin-like 2 proteins of BNM 214 and BNM 216, compared with the predicted tertiary structure of pilins PilA_XAC3241_ and PilA_XAC3240_ of *X. citri* subsp. *citri* strain 306 (Xac306). The online server Phyre2 (Kelley *et al*., 2015) was used for this analysis.


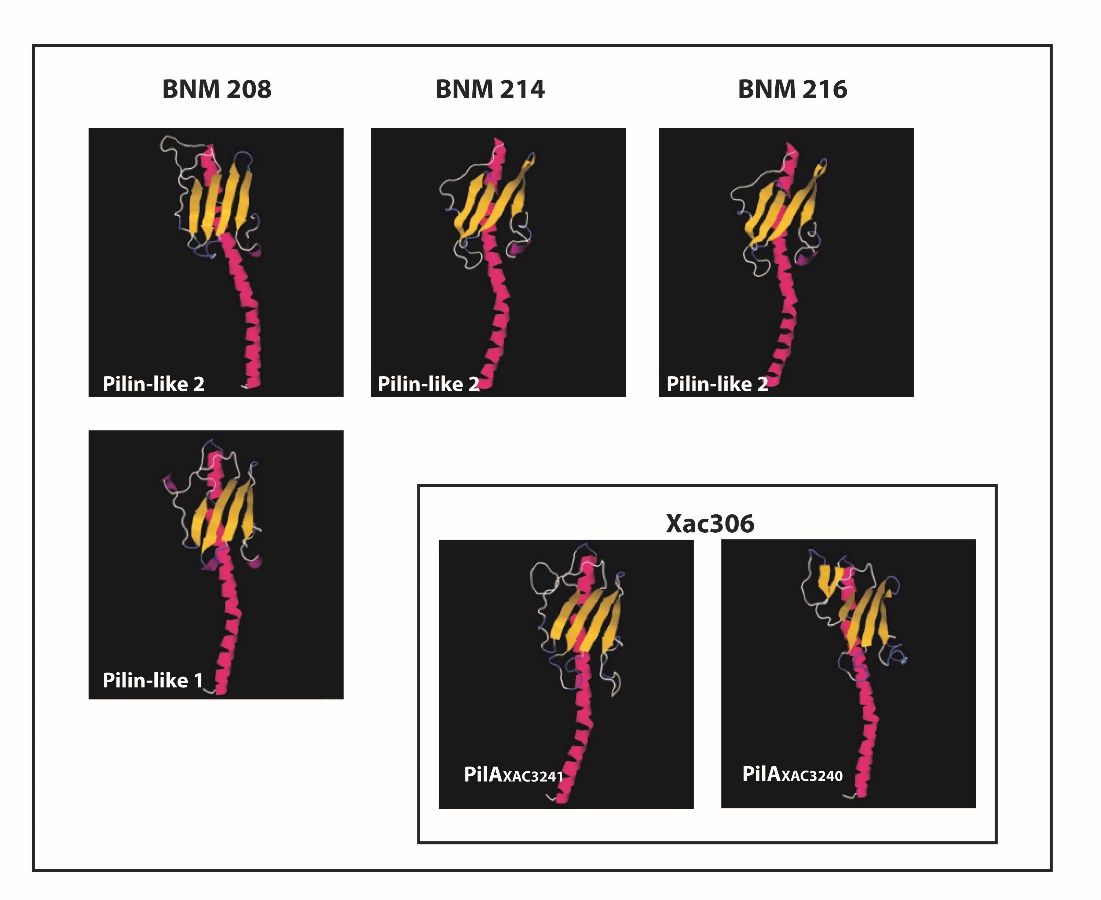


**Supplementary Table S4.** *In silico* analysis and PCR amplification of some genes associated to type IV pili (T4P)

| **Gene** | ***In silico* analysis^a^** | | |  | **PCR amplification^b^** | | |
| --- | --- | --- | --- | --- | --- | --- | --- |
|  | **BNM208** | **BNM214** | **BNM216** |  | **BNM208** | **BNM214** | **BNM216** |
| ***fimT_xac2669_*** | (0) | (-) | (-) |  | (+) | (-) | (-) |
| ***pilX_xac2666_*** | (0) | (-) | (-) |  | (+) | (-) | (-) |
| ***pilE_xac2664_*** | (0) | (-) | (-) |  | (+) | (-) | (-) |
| ***pilW_xac2667_*** | (1) | (1) | (1) |  | (+) | (+) | (+) |
| ***pilA_xac3241_*** | (0) | (0) | (0) |  | (+) | (-) | (-) |
| ***pilA _xac3240_*** | (0) | (-) | (-) |  | (-) | (-) | (-) |

^a^ (1) indicates that the gene was found with a similarity higher than 80 %, (0) indicates that the gene was found with a similarity lower than 80 % and (-) indicates that the gene was not found.

^b^ (+) indicates that the gene amplified by PCR, (-) indicates that the gene did not amplify by PCR. To verify its functionality, primers used to amplify *pilA_xac3240_* were analysed using genomic DNA of *X. citri* subsp. *citri* strain 306 (Xac306).

**References**

- Kelley, L. A., Mezulis, S., Yates, C. M., Wass, M. K., and Sternberg, M. J. E. (2015). The Phyre2 web portal for protein modeling, prediction and analysis. *Nat. Protocols* 10, 845–858. doi: 10.1038/nprot.2015.053
- Felipe, V., Romero, A., Montecchia, M.S., Vojnov, A.A., Bianco, M.I., and Yaryura, P.M. (2018). *Xanthomonas vesicatoria* virulence factors involved in early stages of bacterial spot development in tomato. *Plant Pathol*. 67, 1936-1943. doi: 10.1111/ppa.12905
- Vancheva, T., Bogatzevska, N., Moncheva, P., Lefeuvre, P., & Koebnik, R. (2015). Draft genome sequences of two *Xanthomonas vesicatoria* strains from the Balkan peninsula. *Genome Announc*. 3, e01558-14. doi:10.1128/genomeA.01558-14
- Richard, D., Boyer, C., Lefeuvre, P., Canteros, B.I., Beni-Madhu, S., Portier, P., et al. (2017). Complete genome sequences of six copper-resistant *Xanthomonas* Strains causing bacterial spot of *Solaneous* plants, belonging to *X*. *gardneri*, *X*. *euvesicatoria*, and *X. vesicatoria*, using Long-Read Technology. *Genome Announc*. 5, e01693-01616. doi: 10.1128/genomeA.01693-16
- Potnis, N., Krasileva, K., Chow, V., Almeida, N. F., Patil, P. B., Ryan, R. P., et al. (2011). Comparative genomics reveals diversity among xanthomonads infecting tomato and pepper. *BMC Genom.* 12,146. doi: 10.1186/1471-2164-12-146
- Roach R., Mann R., Gambley C. G., Chapman T., Shivas R. G., Rodoni B. (2019). Genomic sequence analysis reveals diversity of Australian *Xanthomonas* species associated with bacterial leaf spot of tomato, capsicum and chilli. *BMC Genom.* 20:310. 10.1186/s12864-019-5600-x
